# Informing Nutrition Care in the Antenatal Period: Pregnant Women's Experiences and Need for Support

**DOI:** 10.1155/2017/4856527

**Published:** 2017-08-14

**Authors:** Khlood Bookari, Heather Yeatman, Moira Williamson

**Affiliations:** ^1^School of Health and Society, Faculty of Social Sciences, University of Wollongong, Wollongong, NSW, Australia; ^2^School of Nursing, University of Wollongong, Wollongong, NSW, Australia; ^3^School of Nursing and Midwifery, CQUniversity, North Rockhampton, QLD, Australia

## Abstract

This study aimed to provide insights into Australian women's experiences in gaining nutrition information during pregnancy. Individual semistructured telephone interviews were conducted with 17 pregnant (across all trimesters) and 9 postpartum women in five Australian states. Data were transcribed and analysed using inductive thematic analysis. Women valued nutrition information, actively sought it, and passively received it mainly from three sources: healthcare providers (HCPs), media, and their social networks. Women reported HCPs as highest for reliability but they had limited time and indifferent approaches. Various media were easily and most frequently accessed but were less reliable. Social networks were considered to be the least reliable and least accessed. Women reported becoming overwhelmed and confused. This in turn influenced their decisions (pragmatic/rational) and their eating behaviours (“overdo it,” “loosen it,” “ignore it,” and “positive response”). Individual and environmental barriers impacted their application of knowledge to dietary practice. Women wanted more constructive and interactive engagement with their HCPs. This study identified the need to establish and maintain mutually respectful environments where women feel able to raise issues with their HCPs throughout their pregnancies and where they are confident that the information they receive will be accurate and meet their needs.

## 1. Introduction

 Poor nutrition has the potential to negatively impact mothers' and babies' health, contributing to poor maternal and infant outcomes. Adverse maternal outcomes include increased risk of inappropriate gestational weight gain (GWG) [1] which has been associated with higher risks of preeclampsia, macrosomia, and caesarean section [2]. Exposure of the foetus to maternal obesity, diabetes, and unhealthy GWG can increase his/her risk of developing childhood obesity and chronic diseases later in life [3, 4].

While healthy eating is critical for the health of mothers and their infants, many women do not sustain an optimal diet during pregnancy [5]. Some pregnant women's diets lack key nutrients including folate, iron, and fibre [6] or fail to meet the Australian Guide for Healthy Eating (AGHE) for some major food groups (fruit, vegetables, breads and cereals (now grains), and meat (and its alternatives)) [5, 7–9].

To help pregnant women achieve safe, healthy, and balanced diets, insight into the factors influencing their dietary behaviours is important. A number of factors have been identified, including physiological (prepregnancy body mass index (BMI), nausea, and vomiting), cognitive/perceptual (knowledge and attitudes), socioeconomic (income, marital status), and institutional and community factors [10]. Nutrition knowledge has also been found to influence pregnant women's dietary behaviours [10–12] and dietary choices [13]. Lack of relevant nutrition knowledge can be a barrier to a healthy diet [14] and appropriate use of supplements, particularly folic acid and iron [15].

Earlier quantitative research has reported on the influence of knowledge, attitudes, and motivation on pregnant women's adherence to dietary recommendations [9]. Interestingly, the majority of women showed high levels of motivation and confidence in their ability to achieve a healthy diet and understand dietary recommendations but were found to have poor knowledge of and poor adherence to dietary guidelines [9, 16] and GWG recommendations [17]. Such women may fail to seek accurate information from their healthcare providers (HCPs). HCPs also may miss opportunities to provide dietary information and support as they may incorrectly assume such women were already knowledgeable [9].

Understanding women's experiences in gaining information and their perceived needs for this information are important aspects in identifying gaps in knowledge and provision of suitable support to meet their needs. The World Health Organization [18] identified that involving women in decision-making and considering them as active participants in optimising their own health are essential components of high quality antenatal services. Thus, in the first instance, it is important to find out from the women themselves their specific needs with regard to nutrition communication and their ideas on how to develop effective strategies and care that promote healthy diet related behaviours [19].

There is limited published research on the experiences of women gaining nutrition-related information during pregnancy [20]. Pregnant women have reported receiving sparse, “general” food and nutrition advice from their HCPs, with provision limited to when health problems or symptoms occurred, or mainly focused on food safety [21–26]. Szwajcer et al. [24] reported that midwives made an effort to build rapport with pregnant women by being friendly, complimentary, confirmative, and supportive; however, providing nonindividualised information deterred women from using it. In many cases, women reported navigating between different sources to seek information [21–23, 27]. Using nonreliable sources of information, such as online resources, has been found to significantly increase women's confidence levels regarding making decisions about their pregnancy [28]; however, confidence does not necessarily equate with having accurate or relevant information or making an appropriate decision [9]. Instead, women may feel uncertain and may adopt nonrational decision-making in dietary management [21, 23, 26]. Finally, data are still scarce in terms of including pregnant women's perspectives on how their experience in gaining information could improve and what support and nutrition care they need to facilitate their utilisation of information and enhance their dietary intake.

The aim of this study was to explore pregnant women's perspectives on their access to reliable and relevant nutrition knowledge and the factors that affected such access. Specific areas for exploration included (1) the sources of nutrition information during pregnancy, (2) women's responses to nutrition information, (3) modifiable barriers that prevented pregnant women from applying nutrition knowledge to their dietary practices, and (4) women's perceptions of nutrition information and appropriate models of nutrition communication during pregnancy.

## 2. Methods

### 2.1. Sampling

The 88 pregnant women who had completed the nutrition knowledge survey in an earlier cross-sectional online study and agreed to be contacted to take part in this study were invited to participate via invitation emails and a follow-up phone call. Of these, 35 agreed to participate. In-depth, semistructured interviews were conducted with them via telephone between May and July 2013. However, data saturation point was reached after 26 interviews, when data collection was ceased. Hence, the subsample size was 26.

### 2.2. Data Collection

All women were interviewed individually by the primary investigator using a semistructured interview guide (Table 1). The interview guide was reviewed by a panel with expertise in midwifery, maternal health, and public health nutrition. The order of interview questions was flexible to reflect the participants' varied experiences and additional prompting questions were used.

### 2.3. Data Analysis

All interviews were audio-recorded and transcribed verbatim. The length of interviews ranged from 12 to 46 minutes. Transcripts were analysed using thematic analysis. The primary investigator read the transcripts multiple times and compared them with the audiotapes to ensure the accuracy of transcription and to gain greater familiarity with the data. All researchers independently generated formative codes for seven transcripts and discussed any code discrepancies until consensus was reached. The primary researcher then completed the coding for the remaining transcripts. The quotes that best represented the themes were selected for reporting [29] and pseudonyms were used to ensure confidentiality. Throughout the analysis, data were constantly reviewed to ensure the themes reflected participants' narratives.

### 2.4. Ethics Approval

The study was approved by the University of UOW Human Research and Ethics Committee (HREC) (ethics approval number: HE12/296; approval date: 28 September 2012). The South Eastern Sydney and Illawarra Area Health Service, South Western Sydney Local Health District (Campbelltown/Liverpool hospitals) also approved the study utilising the National Ethics Application Form (NEAF).

## 3. Findings

### 3.1. Participants

A summary of interviewees' characteristics is presented in Table 2. The twenty-six pregnant women who voluntarily participated in the telephone interviews had anonymously completed a nutrition knowledge survey [9]. For all but one participant, English was their first language (*n* = 25). The majority of participants were residents of New South Wales (*n* = 19), were pregnant at the time of the interview (*n* = 17), were aged 30 years or more (*n* = 17), and had a university degree (*n* = 19).

A small number of the women had consulted a dietitian after being diagnosed with gestational diabetes mellitus (GDM; *n* = 4) or prepregnancy food allergy (*n* = 1). One did so as a voluntary participant in a hospital study, another privately for dietary management during pregnancy. Four interviewees were vegetarian.

### 3.2. Women's Experiences of Gaining Nutrition Information

For most women interviewed, the health of their babies and themselves had a high priority. Eating healthy foods and knowing about nutrition during pregnancy were equally important.

Gaining nutrition-related information was an interlinked, fluid process of passive reception and active seeking of information from multiple sources. Almost all looked for information as soon as pregnancy was confirmed. In most cases, active information seeking would begin independently by navigating different sources. Most frequently used were the Internet and books. Women also sought information from their HCPs. A few spoke with family members and friends, especially friends with children, to crosscheck a particular issue. However, women would not place any special value on information from their social environment unless the person providing the information was qualified as a health professional or, in one case, one whose views resonated with the woman's personal beliefs. Women also passively received nutrition-related information from their HCPs as a part of antenatal care and from their social environments. In the following section, the process of gaining nutrition information from different sources is described in detail.

### 3.3. Sources of Nutrition Information

Most of the pregnant women reported they had received some level of nutrition advice during their antenatal care, which varied in terms of topics, format, clarity, and adequacy.

### 3.4. Healthcare Providers (HCPs)

The main HCPs for women in this study were general practitioners (GPs) and/or midwives and in few cases obstetricians and gynaecologists. Most interviewees appreciated HCP-provided nutrition information and considered such information as useful. Written information (brochures and/or leaflets) from their HCPs was considered useful as it was deemed factual and served as a reminder of what they already knew. Being supplied with test results helped generate a sense of reassurance. For example, when their HCPs provided their blood test results, they regarded this as proof of good nutrition. Positive comments on their behaviour, for instance, amount of weight gained, were also valued as reassurance.

Most interviewees indicated that their HCPs were the most trusted source of nutrition-related information. Doctors and obstetricians were reported as the most trusted sources, followed by midwives and then any government-sourced scientifically based information.

While the majority of interviewees considered their HCPs as trusted sources of information, they also reported that their HCPs failed to meet their needs for nutrition-related information during pregnancy. The information they had received was inadequate, limited in content, and general in nature. Even the general information that had been provided the women found impractical and difficult to apply.

None of my doctors, like my obstetrician or my GP, neither of them really have said specifically what to do. They just sort of said, “Oh you know eat a healthy diet”, but they haven't said what …. (Sophia)

The women constantly looked for more comprehensive and practical information using other resources.

Little time was allocated by HCPs for discussing nutrition-related issues or to clarify issues. Approximately one-third of the participants indicated they would discuss their concerns with their HCPs if there were time or if the concern arose near their appointment; otherwise, they made use of other sources. Even when the women asked questions or sought further details, they reported that their HCPs most often gave them a short answer or referred them to someone else who often did not provide further information. Such experiences were clearly frustrating for these women.

… If you've got questions, they will either quickly answer them and won't give you an opportunity to maybe clarify stuff …, or they will send you somewhere else and then you've got to go and sit there for another hour and a half to wait to see someone else to get a two-minute answer — … that kind of thing kind of really irks me. (Amelia)

At different stages of pregnancy (either at pregnancy confirmation or during antenatal appointments), HCPs often handed out pamphlets, brochures, and booklets published by government bodies but without any discussion with the women. In a few instances, participants mentioned that their HCPs voluntarily went through the provided resources briefly and kept checking on the women's knowledge and understanding of the information.

…the midwife like gave me a pamphlet — I remember she circled things of … in regards to listeria and that what was dangerous — …. There was that pamphlet, otherwise no, it was supposed to be my own research. (Emma)

In many cases, women reported that their HCPs were “not forthcoming” in providing nutrition-related information. HCPs expected women to ask for such information. This could be problematic for many women, especially for primigravidas and those who were uncertain about what questions to ask. Time constraints meant the women did not have time to ask or simply forgot to do so.

Wasn't helpful … probably just because the doctor [was] sort of busy. Unless you wrote down the questions, you'd walk in and forget, and there'd just be a lot of other questions. (Ava)

Some women reported that they had initiated conversations about nutrition-related topics but their HCPs appeared unconcerned. In most cases, the HCPs were medically driven in the advice they provided. Only when women had medical conditions or health-related issues did the HCPs discuss and provide some specific and personally relevant information.

They [HCPs] also sort of looked at my history and saw that I had deficiencies in iodine before, and Vitamin D, so they sort of gave specific information, specific to me, on what I could do and what supplements were available…. (Allison)

As part of antenatal care at the study sites (public hospitals), women saw a number of HCPs, usually not the same one at each visit, each of whom had different interests and views about maternal nutrition issues. As a result, some of the participants reported receiving conflicting advice. Conflicting information increased women's confusion about nutrition information.

…when I had to switch to the antenatal, like the midwife clinic, that's when I started getting a lot of confusion because stuff that she [obstetrician], that we'd worked out with her that was working for me, they sort of didn't always agree with… and it's all very conflicting and everyone has a different idea about what's good…. (Emily)

In addition, some women complained about receiving inaccurate and impractical advice that did not take into consideration their specific food preferences (often ethically or religiously based).

I'm a vegetarian, so someone saying that I need to eat more meat to increase my B vitamin and iron intake isn't useful. (Olivia)

While their HCPs often monitored GWG, they rarely provided deeper counselling or advice about the GWG recommended range or dietary management. It became apparent through the interviews that HCPs' advice was mainly focused on what not to eat and on taking pregnancy-specific supplements. Almost all HCPs asked women to take a pregnancy-specific multivitamin and some of them checked women's adherence to these supplements. The women complained of the inadequate information provided, mainly in regard to food sources of these nutrients, their importance, and the risks associated with their deficiency.

No-one has ever given me advice regarding food; it's always just take a tablet or take a supplement… I think that's really lacking. (Zoe)

In spite of the identified limitations in HCP-provided nutrition advice, more than half of the participants stated that they were generally satisfied with the information they received. This satisfaction, however, is in part attributed to their taking personal responsibility for information seeking. They expressed confidence in their ability to gain the required information from other sources, for example, socially, if they had a scientific background or higher level of education (e.g., postgraduate) and accessed gestational diabetes mellitus (GDM) management recommendations. A multiparous woman also felt satisfied as she thought she had an existing reasonable level of understanding.

Yes, I am [satisfied], even though she [HCP] hasn't said much… I guess so, I know where to get information. (Olivia, immunologist)

### 3.5. Popular Media

Acknowledging the importance of healthy food for the baby and accepting responsibility for obtaining the desired information, more than three-quarters of participants actively sought information from different sources, often in response to unmet needs. Some sources reinforced accurate health messages, but others had a lower degree of reliability and provided inaccurate, confusing, and conflicting information. Rather than informing them and helping direct their decision-making, the amount of conflicting information impeded it. The Internet was the main resource used by almost all women, followed by books on pregnancy.

Most women sought nutrition-related information as soon as they knew they were pregnant. For every question, concern, and doubt, all women reported they started the journey of seeking information by “googling it.” Women reported using the Internet to gain information about what to eat for a healthy pregnancy, further details about high-risk foods, and recommended GWG.

The women's preference for the Internet was attributed to a number of factors including anonymity, ease of access, the apparent veracity of much of the material, and the fact that it was straightforward and user-friendly. One participant considered that online research reinforced what she knew from her previous pregnancies.

[B] because I'd been having such a big gap between kids, I had forgotten a lot of the stuff that you weren't allowed to eat, and so checking online was a good thing because it kind of reinforced things. (Amelia)

On the other hand, some women expressed some concern about the Internet as they found some information inaccurate, inconsistent, not culturally relevant (e.g., American-based), unhelpful, and confusing. Some participants were worried about inaccurate information. Emily gave the example of a friend who was influenced by inaccurate information accessed online and questioned listeria risks.

…[She] found this website that she really liked and she was following it to a tee but it was sort of saying, you know fifty years ago, people didn't watch out for listeria and all that sort of stuff and all the babies were born healthy so she was eating a completely different diet to what I was… enjoying alcohol, and she didn't really care about soft cheeses and stuff like that…. (Emily)

Other participants had been exposed to international dietary practices and were confused by variation in dietary advice between countries.

I just knew the listeria: they said it was a dangerous here but then I know in France…,… they don't say things like that, they also … say having a glass of wine once a week or whatever is fine. (Zoe)

Primiparous women were more involved in the process of information seeking and used books more often than multiparous women.

I did buy a book. … “What to Expect When You're Expecting”,… when I was pregnant with my first and I relied on that quite a lot to get information. (Allison)

The perception of the trustworthiness of the information women found on the Internet or in published resources (books, magazines) was affected by how they assessed the information. Most women would not follow information that appeared to be a personal opinion or came from personal blogs and forums. The main way of evaluating the information—adopted by more than half of the women—was cross-checking and comparing information. Most interviewees did not check the authors of sourced websites but compared the information against different websites and took notice of the information that appeared most consistently.

I checked a couple of sites that come up on the Google and just kind of cross reference to make sure that they were all saying the same sort of thing but that's [it] basically. (Amelia)

Only one in four women compared the Internet-sourced information from nongovernment sites against information on government websites. However, the material they sourced was often from sites written by doctors, or from scientific literature.

I guess I first compare … the different sources, like I'll go by my doctor's booklet first because I trust the information that she's given me, but I get… more detail from [internet] sites … and if it's saying the same thing as what the booklets given me but more detail on top of that, then I usually go by that. (Emma)

Larger, well-known, and most frequented websites were also trusted by about one-quarter of interviewees. Their personal knowledge and “common sense” contributed to the eventual decision made.

I probably don't check the author of the individual article on the website I just figure if it's one of the bigger, more well-known websites. I sort of trust that its reasonably well vetted and I don't believe exactly what I read in it, I kind of feel confident that I kind of mix that with my own knowledge or my own common sense. (Lily)

### 3.6. Social Network (Friends and Family Members)

Using family and friends as a resource was considered the least reliable and was least frequently relied upon. Women indicated that they exchanged some reading materials (books) to provide social support and would only chat informally with a friend regarding pregnancy related information.

I know I had a chat with other friends who have been pregnant about their pregnancies and things and what they ate and what they found… but I didn't really speak to them [to get information]. (Charlotte)

One participant sought information from a friend who she assumed was knowledgeable as she “*is a nurse … I will ask her. She's a good source of information” * (Zoe). Another participant indicated that she trusted her friends over HCPs as they had experienced the same situation first-hand, adding that the science did not suit everybody.

I definitely I would trust my friends more … because the things they told me once they experienced, so I mean I know some information comes from GP or midwife will be more … scientific evidence or something … but … the scientific things … are not suited to everybody that's why I trust my friends more. (Madison)

Some participants expressed a concern about being overwhelmed by the amount and the different sources of information being accessed, which made it hard to use information.

### 3.7. Women's Responses to Nutrition Information

Participants reported that they were eager to not cause any harm to their unborn babies and to nourish them well. Most reported altering their food intake to some extent. However, the extent depended on their levels of confidence and knowledge and the availability of nutrition care and support. The levels of confidence and knowledge also influenced the women's information seeking behaviours.

The most frequent dietary changes reported were to avoid high-risk foods and take pregnancy supplements following HCP advice received during antenatal care, verbally or written (booklets and pamphlets), and through information accessed online, mainly from (NSW) government websites. Some participants made no further alteration to their prepregnancy diet as they were confident about adjustments they had made prior to pregnancy to ensure the adequacy of their diet.

I don't really need to make big changes. My biggest concern is usually getting things like folic acid and iron and enough protein but that I have, I make adjustments to how I eat all the time to accommodate that and being pregnant doesn't really change for me to optimise the intake sort of thing. (Olivia)

Women were very responsive to advice/information when it resonated with their beliefs and/or personal preferences. Most participants made a positive effort to follow professional advice when they had adequate advice and support, even when recommended changes were against their personal choices. For example, one participant abandoned her gluten-free diet (a personal choice) to best manage her GDM; and another participant, a vegetarian who was iron deficient, started to include meat in her diet after failing to tolerate supplements.

Participants reported dealing with the volume and sometimes conflicting information in different ways. Motivated by knowledge of one's limitations, participants sought accurate information, but depending on the sources they had accessed, their response varied. Where women accessed unreliable sources, some became uncertain and they were still aware of their ignorance. Others lost their sense of ignorance and felt confident that they had become knowledgeable, even when they were not (as evidenced by their misperceptions). In the latter instance, participants lost interest in seeking information, believing that they were knowledgeable. For example, one participant did not recognise that her knowledge of listeria risk was incomplete. She was confident about her knowledge and was not interested to learn more. Such a state can lead to dangerous practices.

I seldom worry about food safety because I always think of things like listeria as being more often a meat problem than a veggie problem. (Olivia)

When women had accessed reliable information sources, a positive result was evident and they expressed confidence in their knowledge and capability of making a sound decision and taking appropriate action. For example, an interviewee conducted formal research about the omega-3 fatty acids and became aware of their importance for her baby's health and acted upon what she has learnt.

I've actually done Omega 3 research, so that's...... something that I was already, like well-versed in… I changed a flax oil supplement or add walnut oil to stuff to the Omega 3 that I took in because the nut oils don't have as much DHA. (Olivia)

Another interviewee knew that she did not know how to manage her GWG and at the same time enjoy healthy food. First, she made a personal effort to gain information but with no success. At that point, she sought help from a dietitian and was very happy with the support provided. This woman not only changed her diet positively and managed her weight gain appropriately but also decided to continue meeting with the dietitian after the birth to gain breastfeeding information.

I didn't know how much [weight] I should gain and when I should gain it. I was concerned [about] what I should eat …. That's why we went to source out somebody who specialised in it… I gained seven kilos in total… so when I had the baby I was actually a little bit lighter than when I started [overweight] … I thought she [dietitian] was wonderful and … got an appointment with her six weeks after birth I'm going to talk to her again about breast feeding nutrition. (Mia)

Women who actively sought information from different sources could become confused in terms of their knowledge but subsequently were pragmatic in making decisions about their dietary intake (because they still needed to eat and feed their family). Mostly, they would depend on “common sense” and their assessment of the risk to filter out the information and to justify their decision before applying it to their diet. Some women applied very strict rules on themselves and avoided any food where they doubted its safety. Others decided to consume food of dubious safety, justifying their action by saying that it was not harming them and/or their babies. This was more prevalent when the food was a “*favourite*” or commonly consumed item.

I have found it personally really hard to cut things out during my pregnancy, to let go of foods I've really, really loved. I've also done a risk assessment, like I will still have eggs that are slightly runny, not raw … because I feel that, you know, that doing that occasionally isn't the worse thing in the world. We have our own chickens. I'm cooking eggs that we've grown and things like that. (Aubrey)

Very few women made food choices without “justification.”

### 3.8. Barriers to Applying Knowledge

A number of factors, environmental and individual, were identified by participants as barriers to the application of knowledge. Environmental barriers identified by participants included their HCPs, family members, friends, transport, and geographical constraints.

Interviewees reported a lack of adequate information from their HCPs as the main barrier to healthy eating (as described earlier). A few identified a lack of family support and poor access to foods as barriers to healthy eating, particularly when they have been advised to eat something that they knew their family/husband would not eat. Not a direct barrier but perceived as unhelpful could be friends' comments on the woman's eating practice.

I found other people's opinions of what I was eating unhelpful, like friends and work colleagues..... some people would be like, oh you realise you shouldn't be eating that? And I'm like,…, I am aware but it's[ham sandwich] toasted, it's been heated. (Aubrey)

Common individual barriers to healthy diets pertained to a lack of nutrition knowledge and cooking skills and time and cost constraints. Other less frequent factors identified included physiological factors (cravings, nausea, and tiredness), personal preference for certain foods, (self-reported) laziness, and tolerance of risk taking.

[A] lack of knowledge about how to prepare healthy food... I think for most people it's intimidating to prepare fresh food, like they don't know what to do with it. (Olivia)

Time was a major constraint for full-time working women and women with family commitments.

… This is my second pregnancy, because I've got a toddler, eighteen months old – time to cook meals and to …sit down and eat properly and make sure you're drinking enough water, [is difficult] because you're busy all the time. (Cara)

Other barriers included the belief that eating healthy would be more expensive than eating less healthily and the use of “scientific language” to communicate nutrition knowledge. A few women found it difficult to translate the recommendations into practice, especially when the information was presented in grams not in portions or serving sizes.

### 3.9. Women's Perceptions of Their Needs for Nutrition Information and Appropriate Support and Nutrition Care

Most participants believed that knowing about nutrition was important and they had a desire to nourish and protect their unborn babies.

They expressed their desire to utilise provided information to the benefit of their babies' health and their own. Interviewees were interested in receiving further advice and information about healthy eating during pregnancy and what that could entail—including how they can avoid “eating for two”; what type of food to increase, by how much; and what the recommended portion size was.

Probably what would have been good would be a guide of how much, what sort of portion sizes and … it would be more of a case of me wanting more information on what foods to include, to boost those, those important nutrients…. (Cara)

Other specific information requests related to an optimal vegetarian diet during pregnancy, omega-3 fatty acids and safe fish options, including dispelling the conflicting messages about the type and amount of fish able to be safely eaten.

Interviewees expressed the opinion that nutrition advice should focus on foods as a whole and not be fragmented into instructions to take certain nutrients. Their preference was for information to be presented as multiple food choices to meet the requirements for pregnant women and their babies' health.

If you can highlight a food that's both high in iron and in calcium or a food that's high in B vitamins and beta-carotene or whatever, like, if you could give people information about what types of foods meet several of their nutritional needs instead of just saying, “You need to take lots of folic acid.” (Olivia)

For the majority of women, HCPs (including obstetricians, GPs, and midwives) were the personnel most preferred to provide nutrition-related information. Some proposed involving dietetic services as part of antenatal care.

… If they had like a dietitian visit as part of your ante natal visits… For … your first ante natal visit [to] involve a 15 minute consult with a dietitian that would probably be useful. (Olivia)

Pregnant women particularly wished that their HCPs would be more proactive and forthcoming in providing them with nutrition-related advice. They wanted their HCPs to initiate conversations about nutrition which would help them raise any question they may have and encourage them to think about the issue. Women felt that providing them with information in a written form alone was insufficient. They suggested that HCPs take it one step further and try to engage them in a discussion.

… a chat about it would certainly help and it would raise the questions initially but then you could start to think about it a bit. (Josie)

The interviewees suggested that their HCPs could provide them with information that was related to women's more frequently asked questions or direct them to other reputable information sources. They considered this to be one way to avoid lost opportunities for receiving nutrition-related information that may otherwise result from many women not knowing what they should be asking.

…frequently asked questions or something like that from other previous patients,… that they could probably keep hold of and give that information out or may just direct [us] to other information that's already available... there's so many different ones [websites], maybe just sort of suggesting, “Okay look, this is a really good one to go [to] that's like reputable.” (Ava)

As most felt overwhelmed by the volume and often conflicting information that they had sourced, they desired specific guidance to follow. Interviewees wanted not only to receive advice from their HCPs but also for the HCPs to help them identify the exact guidelines (extracted from authoritative sources) that the women needed to follow.

.. there's so much of it,… I can get a bit overwhelmed by how [much] there is, the different sources of it,… [I]t probably would have been helpful if my GP had … given me a brochure … with the authoritative source that said, “Right, this is what you have to do and these are the portions you need.” That would be quite helpful. (Sophia)

A number of interviewees indicated a specific interest in linking advice on healthy eating to more information about recommended GWG and some practical ideas on how to combat excess weight gain and cravings.

… I just think generally there should be more information out there about… weight gain being tied to… your BMI. … From what I understand, there is quite a variation depending on what your starting weight was, but there should be more awareness of if you're overweight to start with, then you don't want to be putting on 15 kilos. (Abigail)

For many women, confusion about high-risk foods needed to be dispelled and risks very clearly spelt out. Interviewees suggested that HCPs provide alternative ideas where possible so that women could enjoy safely eating the foods that they loved.

What foods to avoid with your listeria risk would be really handy and spelling it out very clearly. But ways you can still enjoy foods you really like,… so you're not cutting out all this food that you love. (Aubrey)

Most interviewees indicated that they were often very busy with either full-time employment or family commitments and, hence, they showed a strong preference for receiving simple, easy-to-understand. and practical advice that fitted their active lifestyle, including simple nutritious food recipes or appropriate menu planners. One woman suggested that some meal plans and recipes be added to the NSW Food Health Safety Guide, a resource which was positively commented upon by most interviewees.

I guess, because the NSW Food Health Safety Guide is good but then having more, like ideas of plans for meals to help, like suggestions for breakfast … certain cereals may be better than … others because they might have more things in them for when you're pregnant. (Charlotte)

Time for providing nutrition information and that information's timeliness were identified as important. Women indicated that the current length of HCP appointments needs to be increased to accommodate more discussion about nutrition. All preferred to receive this information right at the beginning of their pregnancy, immediately after they tested positive for pregnancy.

The preconception period was also suggested by some participants as a suitable time to receive nutrition information, given the importance of healthy eating on foetal development (as well as immediately following conception itself). Some interviewees suggested that the provision of nutrition advice be divided into trimesters as this would help women not become confused by receiving too much information at once.

[I]t needs to be broken down into sort of trimesters … you do need different types of nutrition throughout the pregnancy … [and] because if you're trying to read information all at once you get a bit bamboozled. (Emily)

The concept of written materials was not rejected but the content and language would need to be carefully gauged. Alongside verbal advice from their HCPs, interviewees indicated that they would prefer to receive the same information in written form to use as a backup. This needed to be presented in an appealing way, using more pictures and colour, dot points, or lists. The women indicated that they wanted advice about what they needed to do, with brief explanations of the reasons for any recommendations, and that alternative options needed to be offered (e.g., aligned to cultural and ethical eating patterns). They were emphatic that information should be widely available and freely accessible.

While some indicated that they would be interested in attending antenatal education groups, most would prefer to have an interactive educational resource including online resources or health “hotline” services when they were looking for answers to specific questions. They preferred personalised advice. One interviewee suggested the following:

…some kind of phone service that you could ring up for some information that would be, that would be good. …, I don't think people would tend to sort of book in to a clinic type service unless there were really a major issue with diets. (Abigail)

A few interviewees were interested in receiving practical lessons to develop their skills in healthy food shopping.

## 4. Discussion

This study is the first of its kind in Australia. To our knowledge, this is the first study to conduct in-depth analyses of Australian pregnant women's accounts of their experiences in gaining nutrition information and subsequent dietary change. Limitations in the provision of nutrition information and support by HCPs were identified, together with potential negative ramifications of this, including confusion, acting on inaccurate information, and dismissiveness of scientific advice. As women still needed to feed themselves and their families, they made pragmatic dietary choices, with potentially negative consequences. Key concepts of women's confidence and competence in relation to nutrition knowledge were identified. The results of the study provide valuable insights that will inform antenatal practices and contribute to better health outcomes for mothers and their babies.

**Figure 1:**
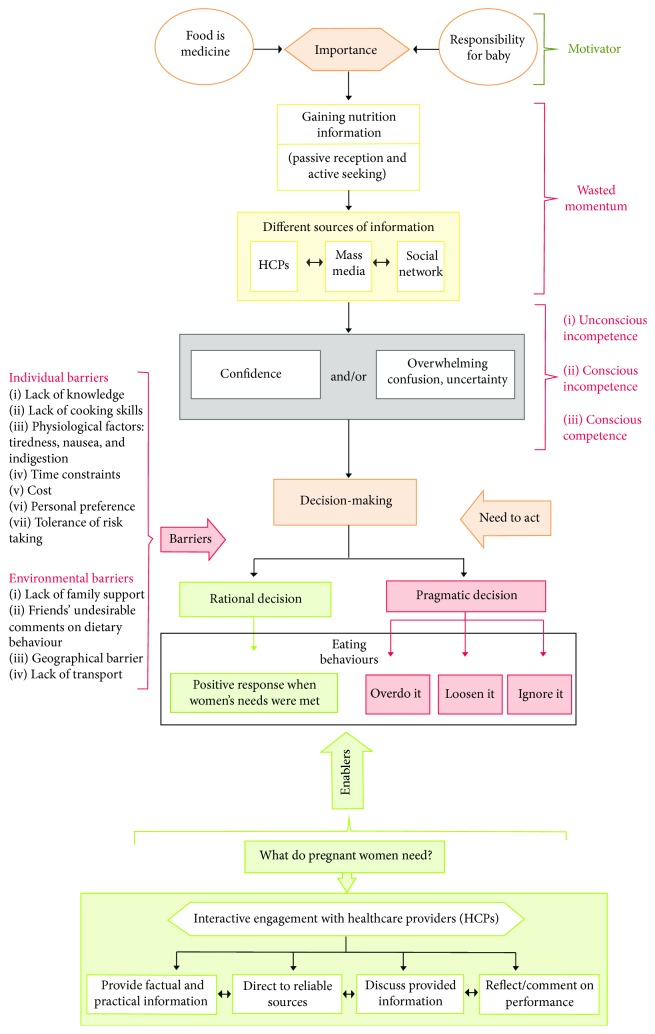
Framework of women's experience in gaining nutrition information and its influence on their eating behaviour as well as the reported barriers and enablers for healthy eating.

The study provides a framework that describes women's experience in gaining nutrition information and its influence on their eating behaviour as well as the reported barriers and enablers for healthy eating, as summarised in Figure 1. The findings highlight pregnant women's awareness of the importance of healthy eating both for themselves and for their unborn babies, confirming the results of an early study [9]. In line with previous studies [14, 27, 30–33], most interviewees valued nutrition information and this prompted their nutrition information seeking behaviour. Szwajcer et al. [33] argued that pregnancy can lead to optimistic outcomes for the future health and nutrition behaviours of women and their families. Thus, pregnancy was an ideal time to promote healthy eating.

Nutrition advice from HCPs (obstetricians, doctors followed by midwives) was especially valued and trusted by pregnant women, although its provision was considered insufficient and not a priority for healthcare support. The valuing of HCP information was consistent with previous research [22–24, 34]. The limited time allocated by HCPs to discuss nutrition-related issues and their lack of active engagement in information provision were consistent with previous research that identified that HCPs possessed limited skills and training to advise pregnant women about nutrition [21, 22, 27]. In addition, the limited and general nature of the nutrition-related information provided and the inconsistency in advice between different HCPs have also been identified previously [21, 27, 34].

Women's concerns regarding the limited quantity and conflicting nature of nutrition-related information provided by their HCPs had several consequences. Women became overly reliant on and used sources like the Internet and books to meet their needs [21, 27, 35]. Such (particularly online) sources advertised and promoted non-evidence-based dietary practices, highly controversial stances on dietary issues, and exposed women to practices and advice that varied depending on the information's country of origin [21]. The results of this study identified that pregnant women lacked the necessary skills to accurately evaluate online information. Even highly educated women's evaluation of information was not always based on its scientific credentials. Selection of nutrition-related information in many cases was often swayed by the women's views and practices. The acquired inaccurate information had the potential to negatively influence the women's dietary behaviours if they acted upon it.

Support for women to develop the skills to judge nutrition information is required. HCPs, such as midwives, are considered to be the authorities, the ones with “the ‘correct' information to deliver” [36]. Being exposed to such information with limited or no skills and in the absence of any obvious guidelines from an authoritative domestic source may result in several consequences, including increased confusion and uncertainty, increased misperceptions, or a growth in trust in non-evidence-based sources. This in turn may lead women to question evidence-based guidelines. Others have also identified that, without proper guidance, information on the Internet can be harmful, confusing, and overwhelming [37].

Women's confidence in their ability to source information varied. Women's competence in different knowledge areas also was variable, both in terms of translating advice into practical food preparation and also in being able to judge their own levels of knowledge. Misplaced confidence in their level of knowledge may impact the effectiveness of their interactions with HCPs.

In this study, women were both motivated and confident in their search for information. According to Lagan et al. [28], women's confidence levels regarding decision-making during pregnancy increased after Internet usage. This can be problematic, depending on the quality of the resources accessed. Women's increased confidence may act as a barrier to effective nutrition education strategies if the information accessed is inaccurate [9], especially if they do not have the ability or the knowledge necessary to distinguish between what was accurate and what was not. As Darwin [38] observed, “ignorance more frequently begets confidence than does knowledge” (as quoted in [39], p. 1121).

Too much or too little information increased the risk of uncertainty and confusion and may impact the translation of women's high motivation for a healthy diet and their practices. Too much information left pregnant women feeling overwhelmed, often with most of their questions unanswered or with answers of varying or dubious value. This may impact the amount of information that could be acted upon effectively [9]. Pregnant women's information seeking experience also was limited in terms of gaining reliable nutrition knowledge, which is consistent with other studies [27, 34].

Most interviewees did not discuss the information they accessed from the Internet with their HCPs, a finding which was consistent with a previous literature review [40]. This was especially the case if they felt confident in the (not necessarily accurate) information obtained and when their HCPs had not been initially forthcoming in providing information. HCPs may not, therefore, be aware of women's possession of potentially inaccurate information or their mistaken beliefs about pregnancy and diet. The HCPs would be unable to (re)address such misinformation unless the women were comfortable about raising the matter with their HCPs and were given the time to do so, and their HCPs had time to respond adequately.

**Figure 2:**
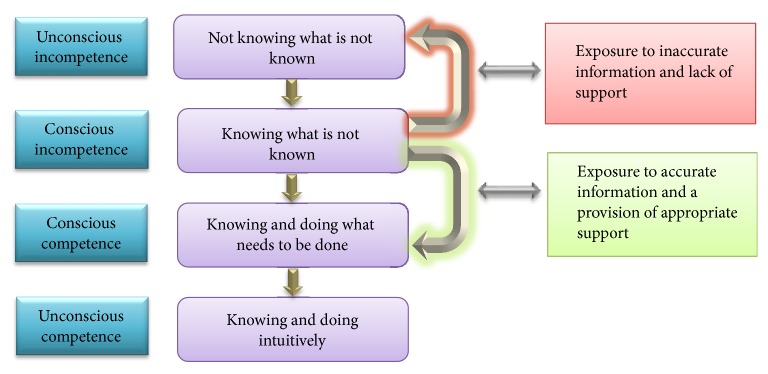
Observed women's perceived knowledge per Conscious Competence Learning Model.

Women's experiences in relation to accessing nutrition information during pregnancy were influenced by their perceived knowledge and level of confidence in their knowledge. Women's perceptions of knowledge revealed links with the Conscious Competence Learning Model (Figure 2), which was developed by Burch in the 1970s and consisted of “Four Stages for Learning Any New Skill” [41]. This model has been used in training in business settings [41] and used in medical education settings [42, 43].

The Conscious Competence Learning Model focuses on two main aspects of individuals' thinking during the process of learning a new skill: awareness (consciousness) and skill level (competence). As is the case in other learning situations, women begin pregnancy with a vast number of “unknown unknowns.” Initially unconscious of their incompetence, women are in a state (or learning stage) known as “unconscious incompetence.” As they become alert to their incompetence, they enter a state of conscious incompetence. The knowledge of their ignorance or lack of skill motivates them to acquire a skill (in this case relevant knowledge). Then, they begin the slow acquisition of understanding and skills, until they are able to consciously utilise these skills (conscious competence). In time, their mastery of facts and decision-making in certain areas will become so automatic that they then work mainly through what is commonly described as “intuition” but in reality is based on past experience and repeated practice. They are then able to seamlessly perform the acquired skills (or apparently effortlessly use the acquired knowledge)—a stage known as “unconscious competence” [42, 44].

Results of this study reflected the first three stages of Burch's model. Women respondents were in different “competence spaces” in regard to different topics. Primiparous women were likely to be “unconsciously incompetent” in regard to some areas, as they did not know what they did not know and thus they were less likely to ask their HCPs about nutrition. Additionally, some pregnant women misperceived their knowledge and did not recognise that they did not have the correct information. Such persons were doubly disadvantaged: they lacked a knowledge/skill but did not recognise that this was the case [39]. Women in this situation may even deny the need for information or its value, which may result in inaccurate conclusions and poor decision-making.

The results of this study identified that individual pregnant women may pass from one competence space to another within the same knowledge area. For example, a woman may pass from unconscious incompetence to conscious incompetence and when motivated by knowledge of her limitations may seek accurate information and then put that information into practice (conscious competence). However, the outcome of learning in the stage of conscious incompetence may depend on the information women accessed and the support they received to confirm the accuracy of the information. If unreliable sources were accessed and incorrect information was obtained, women may regress to lacking awareness of their inaccurate or incomplete state of knowledge (“unconsciously incompetent”). In this study, only a few women were identified as proceeding to the conscious competence stage. They succeeded by accessing continuous support from their dietitian or diabetic educator or by conducting formal research on a specific topic (such as omega-3 fatty acids).

Knowledge acquisition and implementation can be uneven and different stages can coexist in different knowledge domains in a single person. To increase the effectiveness of women's practices, HCPs require skills to identify pregnant women's levels of confidence and competence in relation to nutrition knowledge and provide information and support that matches women's needs.

Interviewees' dietary choices were often framed by pragmatic choices. Unfortunately, this resulted in most cases in inappropriate responses to the information obtained, either by “overdoing it” or by “loosening it” after rationalising the decision, while a very few women chose to not respond to dietary recommendations. “Overdoing it” can be demonstrated by both overrestriction and overconsumption. For example, although women's knowledge in regard to general food safety issues was the highest compared to other nutrition-related domains [16], there was considerable confusion regarding specific details about high-risk foods. Other studies report similar findings [26]. To protect their unborn babies, some women chose to overly restrict their diet. This has been identified as common practice and claimed to be a constituent of the nutrition-related “mothering norms” in which sacrificing favourite foods was preferable to risking any harm to babies and being a “bad” mother [45]. Such restrictions may place a burden on pregnant women and needlessly deprive them of a food they prefer and one that is necessary. Unnecessary food restriction can jeopardise pregnant women's intake of particular nutrients [46]. Overconsumption is a further risk. For example, women can access nutrition messages that encourage meat consumption to improve iron intake during pregnancy but do not know the amount by which to increase their intake, thus leading to intake that exceeds the recommendations [9].

Some women find restrictions very challenging, especially when it interferes with their favourite or everyday foods and where they are unsure of the level of risk posed by particular foods. Here, pregnant women may allow themselves to be a little more flexible and “loosen up” on the guidelines. Women in this study, trying to cope with the guilt that results from loosening the “mothering norm” and trying to avoid acting like a “bad mother,” found themselves needing to justify such deviations from a norm. This echoes the results of previous studies that have reported the need for justification as intense and necessary for pregnant women to legitimise their eating behaviours when their deviance has called into question their ability to provide the ideal prenatal environment for their baby [27, 45]. In most cases, women in our study did not utilise a scientific basis to justify their decisions. Although women still can enjoy their preferred foods by following safe procedures, nonrational justifications can be a concern as women might unintentionally risk their baby's health. On the other hand, a few women exhibited positive dietary changes. This was in response to accurate information that was provided by their HCPs. This emphasises the important role HCPs may play in promoting healthy eating and the importance of their accurately ascertaining and meeting women's needs. HCPs need to improve communication skills to maximise information impact.

Women identified a number of aspects which should be considered in order to improve nutrition communication strategies in antenatal care. Besides the extensively described limited support from antenatal HCPs, a lack of nutrition-related knowledge and cooking skills, time and cost constraints, and physiological factors (e.g., pregnancy discomforts, nausea, heartburn, and tiredness) were the main reported individual barriers to healthy eating. Women also considered a lack of family support, friends' undesirable comments about their dietary practices, and difficulty in accessing a healthy diet (including transport and geographical location) among the environmental barriers to healthy eating.

The women identified several mechanisms that could improve their levels of nutrition knowledge with most relating to strengthening the role of HCPs. In this study, women preferred information that provided medical knowledge, written by health professionals on a scientific basis, reflecting the concept of “authoritative knowledge” [47]. From this perspective, knowledge is considered to be valid and important not necessarily in its accuracy but in its relation to the particular setting [47]. However, in relation to nutrition to prevent misperceptions and deleterious outcomes, accuracy also is important.

The women interviewed preferred to get the information as soon as they knew about their pregnancy. Some would even prefer to learn about the nutrition in the preconception periods. This proposal may reflect the level of education and overrepresentation of health-related qualifications among interviewees.

Women's suggestions for improved communications involved mainly practically oriented knowledge and skills, together with some factual information to understand the relevance and importance of the information supplied. Consistent with previous studies [19, 30, 31], women needed further information, more time (with their healthcare professional) allocated for nutrition communication, and a tailored approach to care provided in an interactive environment that allowed for women's dynamic participation. The main requested information was on general healthy eating for pregnancy in a holistic context, including GWG management, and vegetarian and other dietary plan provisions.

This study identified that HCPs may need to reflect on their interactions with pregnant women, not only in terms of nutrition content and skills but also in terms of building women's confidence and competence in accessing, assessing, and applying such information. Most women also showed a desire to be directed to reliable additional support using interactive approaches via phone technology (e.g., hotline) or interactive websites (e.g., simple and quick recipes, healthy eating guidance). This was believed to provide continuous access to reliable information to meet their immediate need.

## 5. Strengths and Limitations 

The major strength of this study is that it provides a holistic view of the process from women's involvement in gaining nutrition information to the steps they take regarding dietary change. This study explores not only the barriers and the enablers to healthy eating but also the reasons for misalignment between the high motivation (and effort made to gain that knowledge) and the low level of accurate knowledge as well as their ultimately poor eating behaviour. It is this in-depth exploration of women's experience of the process of gaining information and identification of the gaps in their method of gaining information (not just the gaps in the information itself) that makes this study's unique contribution to the literature on this topic.

It investigated not only the influence of motivation and gaining information on women's dietary behaviour but also the influence of individual and environmental factors. This was followed by an exploration of women's perceptions of their needs for nutrition advice, support, and communication during pregnancy. Included are women's perceptions of what were negative factors in their experience of information access and also, from their perspective, what could support better experience and behavioural outcomes. Unlike many studies, information sought from pregnant women was not restricted to a single topic (such as GWG or food safety or supplementation) but was more general and included all aspects of nutrition and GWG.

In relation to the study method, drawing the sample from the earlier quantitative study which comprehensively investigated women's motivation, attitudes, nutrition knowledge, and dietary practice allowed us to collect in-depth information about what characterises women's dietary behaviour. Moreover, the sample was a group of women from five Australian states which allowed us to gain insight into women's experience from different states. All participating women, however, were informed previously about the study aim and this initial knowledge may have created a recruitment bias.

The results of this study cannot be generalised due to an overrepresentation of highly motivated women who were middle-class, highly educated, English-speaking, and very interested in nutrition. Again, the majority of participants were from metropolitan or major regional centres, so the experiences of pregnant women in rural and remote areas may be underrepresented as are the experiences of less educated, working-class, non-English-speaking women. Their experiences could be explored in follow-up studies.

## 6. Conclusion

In this article, attention been brought to the mechanism by which poor dietary behaviour can be a product of pragmatism in decision-making. The article highlights the influence of women's unconscious incompetence and uncertainty and unattained needs on decision-making and draws attention to women's high motivation as wasted momentum when it fails to result in the acquisition of accurate knowledge and adoption of healthy eating practices. It focuses on specific factors that influence women's momentum to adopt a healthy diet.

Limitations in the provision of nutrition information and support by HCPs were identified as were the potential negative ramifications, including women's increased confusion, their risk of acting on inaccurate information or failing to act, and, for a few, an increased tendency to dismiss scientific advice after accessing unsound non-evidence-based but persuasively presented information. Nevertheless, faced with the need to feed their families, women make pragmatic decisions on dietary choices, with potentially negative consequences. The key concepts of women's confidence and competence (the two not necessarily positively relate in relation to nutrition knowledge) were identified. The results of the study provide valuable insights that can inform antenatal practices and contribute to better health outcomes for mothers and their babies.

For most pregnant women, HCPs are at the apex of the reliability hierarchy, which implies a considerable potential for their promotion of healthy dietary behaviour among pregnant women. Being the first and regular contact with pregnant women increases the opportunity to support women to improve their diet and provide them with necessary information. GPs, for example, have been reported to have the potential to provide nutrition information and care that will reduce risk factors and improve pregnant women's nutrition behaviour (and reduce poor nutrition-related outcomes for both the women and their babies) during pregnancy and subsequently. Pregnancy is a critical period for individuals with lifestyle-related chronic disease and for the vast majority of mothers and babies who have the potential to develop such diseases as they age [48].

However, to meet pregnant women's needs, HCPs should consider women's preferences and their need for nutrition advice. Even women who express confidence and superficial satisfaction must be checked on by proactively engaging in discussion to address nutrition misperceptions that might otherwise go undetected.

## 7. Suggestions for Further Research

This study indicates that women are motivated, do value their nutrition during pregnancy, and seek nutrition-related information. They identified a number of barriers and enablers to enhance their experience in gaining such information. Clearly, HCPs have an important role to play in supporting pregnant women and enhancing nutrition care during pregnancy. Further studies are needed to investigate HCPs' views about how they could address women's needs and enhance nutrition care provided to pregnant women. These could be supplemented by research into educational interventions for women as well as the design, content, and timing of nutrition information provided for, as this research has shown, it is not simply a matter of information alone but of when you provide it and how you provide it. It must respond to each woman's needs across pregnancy.

## Figures and Tables

**Table 1:** Summary of key questions used as interview guide.

Key questions
(1) Could you please describe for me what has/have been your main source(s) of information about eating practices and food issues during
pregnancy?
Prompts
(i) Appropriate weight gain or weight maintenance; food safety/listeria; sources of particular nutrients (e.g., iron, folate, omega-3 fatty
acids, and iodine)
Are there other sources of information that your friends have used during their pregnancies?

(2) Are you satisfied with information provided by your healthcare providers?

(3) Can you tell me about this information and how useful was it? (for each source of information, mainly: healthcare providers, media,
and social network)
Prompts
(i) Were there aspects of the information that helped you to make changes to your eating practices? What was helpful/not helpful?
How do you think it could be improved?

(4) Among all these information sources you have quoted, what was the main trusted source for nutrition information for you?

(5) Do you evaluate the information you have accessed? How?

(6) In your opinion, what is/are the barrier(s) that can prevent women from translating their knowledge into eating practice?

(7) In your opinion, what kind of nutrition information service/support would help pregnant women to improve their eating practice
during pregnancy? How would you like that service/support to be provided?
Prompts
(i) Who would be the best people to provide this information/support?
(ii) What are the best ways to provide this information/support? In person? Leaflets? Internet? SMS messages?

(8) Can you please describe particular topics of nutrition information that you think would be useful for pregnant women? What about
particular times during the pregnancy when information would be particularly useful?

(9) Is there anything else you would like to add?

*Note*. The key questions exclude questions used to collect demographic data.

**Table 2:** Participants' characteristics.

Characteristics	Entire sample	%
	Total *n* = 26	
Prior pregnancies		
None	11	42.30
One	7	26.92
Two and more	8	30.76
Stage of pregnancy		
First trimester	1	3.84
Second trimester	12	46.15
Third trimester	4	15.38
Just had a baby	9	34.61
Age		
20–29 years	9	34.61
30–39 years	15	57.69
40 years and above	2	7.69
Marital status		
Married/de facto	26	100
Education		
Some high school or less	1	3.84
High school completed	1	3.84
TAFE^a^	6	23.07
Tertiary education	18	69.23
Household income		
Less than AU$25,000/yr	1	3.84
AU$25,000–50,000/yr	3	11.53
More than AU$50,000/yr	21	80.76
Refused to declare	1	3.84
First language		
English	24	92.30
Other	1	7.69
State of residency^b^		
NSW	19	73.07
VIC	3	11.53
WA	2	7.69
ACT	1	3.84
QLD	1	3.84
Having health and nutrition-related qualification^c^		
Yes	7	26.92
Seen by dietitian and/or nutritionist		
Yes	6	23.07

^a^TAFE: Technical and Further Education; ^b^NSW: New South Wales; VIC: Victoria; WA: Western Australia; ACT: Australian Capital Territory; QLD: Queensland. ^c^Qualification details. These included 4 with allied health qualifications, including 2 nurses, 1 midwife and one assistant in nursing; 1 with a public health degree; 1 with degree in genetics; and 1 immunologist.
